# Emerging role of extracellular vesicles in diabetic retinopathy

**DOI:** 10.7150/thno.92463

**Published:** 2024-02-04

**Authors:** Junyan Zhu, Jin Huang, Yaoxiang Sun, Wenrong Xu, Hui Qian

**Affiliations:** 1 Department of Gynecology and obstetrics, The Affiliated Yixing Hospital of Jiangsu University, 214200, China.; 2 Jiangsu Province Key Laboratory of Medical Science and Laboratory Medicine, Department of Laboratory Medicine, School of Medicine, Jiangsu University, Zhenjiang, Jiangsu 212013, China.; 3 Department of clinical laboratory, The Affiliated Yixing Hospital of Jiangsu University, Yixing, 214200, China.

**Keywords:** Extracellular vesicles, Diabetic retinopathy, Pathogenesis, Diagnosis, Therapy

## Abstract

Diabetic retinopathy (DR), a complex complication of diabetes mellitus (DM), is a leading cause of adult blindness. Hyperglycemia triggers DR, resulting in microvascular damage, glial apoptosis, and neuronal degeneration. Inflammation and oxidative stress play crucial roles during this process. Current clinical treatments for DR primarily target the advanced retinal disorder but offer limited benefits with inevitable side effects. Extracellular vesicles (EVs) exhibit unique morphological features, contents, and biological properties and can be found in cell culture supernatants, various body fluids, and tissues. In DR, EVs with specific cargo composition would induce the reaction of receptor cell once internalized, mediating cellular communication and disease progression. Increasing evidence indicates that monitoring changes in EV quantity and content in DR can aid in disease diagnosis and prognosis. Furthermore, extensive research is investigating the potential of these nanoparticles as effective therapeutic agents in preclinical models of DR. This review explores the current understanding of the pathological effects of EVs in DR development, discusses their potential as biomarkers and therapeutic strategies, and paves the way for further research and therapeutic advancements.

## 1. Introduction

Diabetes mellitus (DM) is a global epidemic with prominent morbidity. According to the 2021 report from the International Diabetes Federation (IDF), approximately 537 million adults worldwide are affected by DM, and this number is projected to reach 783 million by 2045 1. Among DM patients, about 20% present with diabetic retinopathy (DR) at the time of diagnosis, and an additional 40-50% will develop DR as the disease progresses. DR, a microvascular complication of both type 1 and type 2 diabetes, has emerged as the major cause of vision loss 2. Factors such as prolonged duration of diabetes, hyperglycemia, and hypertension contribute to the development of DR. Additionally, pregnancy and puberty can expedite the progression of retinal pathological changes 3. Persistent high blood glucose levels and hypertension lead to vascular abnormality and dysfunction of retinal cells, resulting in cloudy or blurred vision in diabetic patients. At the late stage of DR, sight loss diminishes patients' quality of life and places a burden on healthcare resources. However, the pathophysiological process of DR is highly complex, involving multiple interrelated mechanisms that trigger adaptive changes and dysregulation of retina cells. Regular monitoring and stringent control of blood glucose levels have been shown to prevent the progression of the disease during its early stage. In advanced phase of DR, treatment options include intraocular injections of anti-vascular endothelial growth factor (VEGF) antibodies, laser therapy, and vitrectomy. Although these clinical treatments have shown encouraging results, most DR patients do not achieve significant clinical improvement in vision 4. There is an urgent need to deepen our understanding of the etiology of the disease and develop new approaches for diagnosis and treatment for DR.

Extracellular vesicles are naturally occurring lipid bilayer-encapsulated nanoparticles with varied size, cargos, and origins and can be categorized into several subtypes 5. According to the biogenesis, membranous EVs are mainly classified as exosomes and ectosomes (also termed as microvesicles, MVs) 6. Exosomes are a subset of small particles (30-150nm) formed in internal multivesicular bodies (MVB) and released during exocytosis 7. On the other hand, ectosomes are vesicles ranging from 50 nm to 10000 nm that are directly shed from the plasma membrane of both healthy and damaged cells 8. While apoptotic bodies, one of the most well-studied subtypes of ectosomes, are submicron vesicles only derived from cells undergoing programmed cell death 9. Recently, membranous vesicles produced by migrating cells at the near ends are identified as a novel subtype of EVs and referred to as “migrasome”, whose roles in cellular communication are under extensive exploration 10, 11. It is worth mentioning that there is a growing interest in distinct non-vesicular extracellular nanoparticles (NVEPs) with diameter less than 50 nm, which are known as exomere and supermere 12, 13. Different names have been used in varieties of studies to describe EVs based on the specific biogenesis, diameter, or contents, such as microparticles 14, shedding vesicles 15, and apoptotic blebs 16. However, the International Society for Extracellular Vesicles (ISEV) has proposed the umbrella term “extracellular vesicles” to encompass all types of membrane-derived vesicles. According to the ISEV, these vesicles are further divided into small EVs (sEVs, <200 nm) and medium/large EVs (m/lEVs) based on their particle size (MISEV2018) 17. Therefore, in this review, the term EV will be primarily exploited to refer to all nanoparticles, unless specific name is applicable based on the research context.

**Figure 1:**
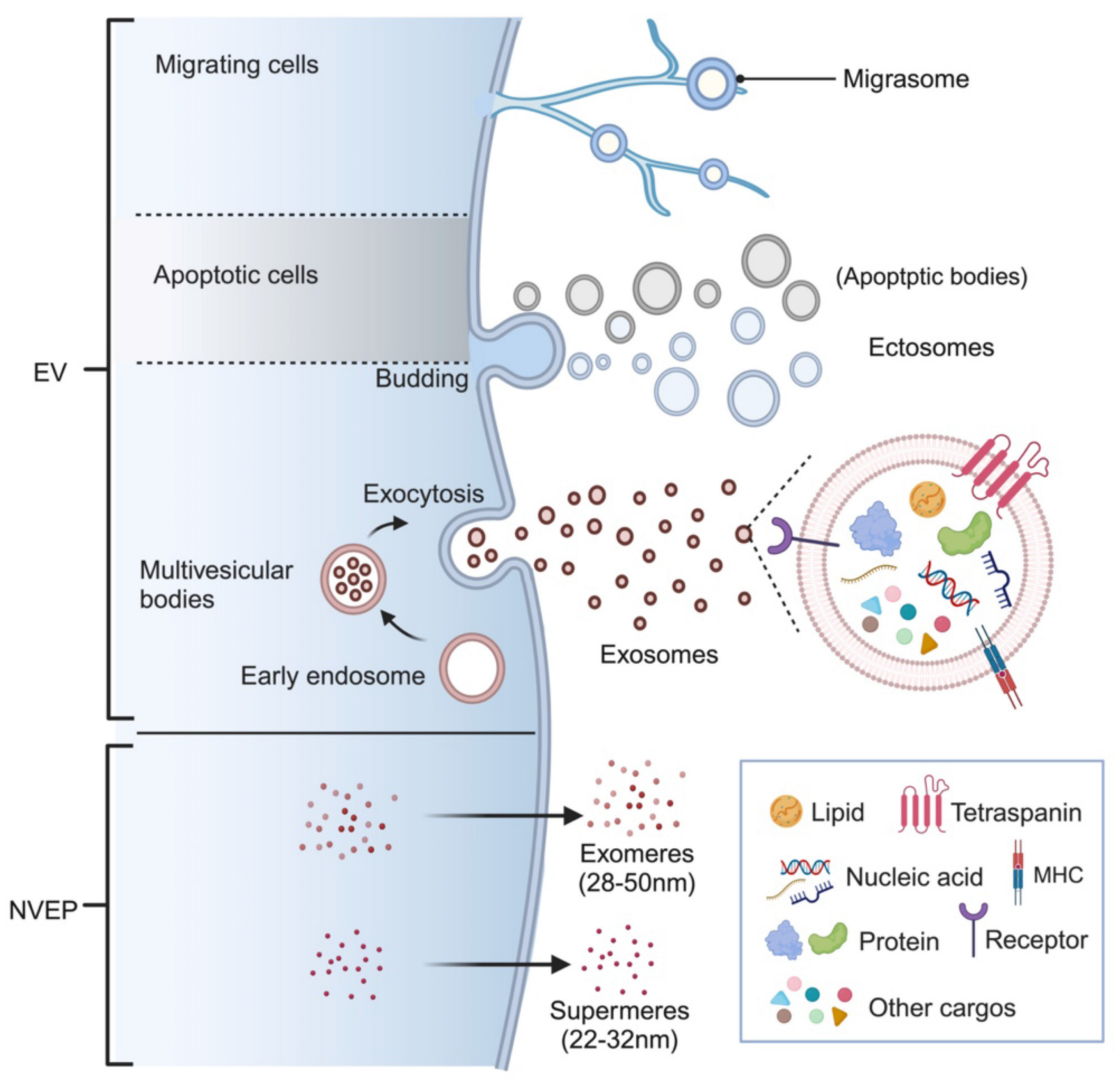
EV classification and biogenesis. Ectosomes are released by shedding from the plasma membrane, while exosomes are generated through the endosomal pathway. Apoptotic bodies, which are released by cells undergoing programmed death, fall into the category of ectosomes. Migrasomes refer to vesicles that formed on retraction fibers of migrating cells. These EVs are enclosed by a phospholipid bilayer and serve as carriers for transporting various cargos, including lipids, proteins, nucleic acids, and metabolites. Interestingly, there are also non-vesicular extracellular nanoparticles known as exomeres and supermeres, which lack a membrane structure but still play important roles in intercellular communication. (Figure created with BioRender.com).

EVs can be released by almost all cells and isolated from the supernatant of cell cultures, a broad range of tissues and body fluids. Enclosed by a membrane, EVs contain a rich assortment of components, primarily including proteins, lipids, nucleic acids, and metabolites. Certain components found commonly in EVs serve as markers for EV identification, such as certain transmembrane proteins (CD9, CD63, and CD81), membrane transport/fusion proteins (Annexins and Rabs), MVB synthetic proteins (TSG101 and Alix), and chaperone proteins (Hsp60, Hsp70, and Hsp90). Additionally, EVs also possess unique compositions specific to their origins, reflecting the physiological or pathological states of the parent cells or tissues (Figure 1). The distinct changes in EV quantity or components make them valuable as disease biomarkers. When taken up by neighbor or distant receptor cells, the cargos carried by EVs can modify their biological functions, enabling cellular information transmitting without direct contact 18. Presently, an increasing number of studies are dedicated to exploring the use of EVs as therapeutic agents and ideal drug delivery carriers due to their specifical biological characteristics 19. The therapeutic benefits of EVs, especially those derived from mesenchymal stem cells (MSCs), have been proposed for a broad range of common or refractory diseases, including diverse retinal disorders 20. In this review, we provide an overview of DR and elucidate how EVs contribute to the progression of DR pathologies. Furthermore, we summarize the potential of EVs as biomarkers and therapeutic agents for DR.

## 2. Development and Pathological changes of DR

As a progressive disease, DR undergoes several clinical stages with increasing diseases severity. Based on the modified Airlie House classification scale as applied in the Early Treatment Diabetic Retinopathy Study (ETDRS) 21 and 2017 position statement from the American Diabetes Association 22, the chronopathology of DR can be clarified into four overlapping phases: mild non-proliferative DR (NPDR); moderate NPDR; severe NPDR; proliferative DR (PDR). Mild NPDR is the earliest stage of the disease, characterized by the presence of microaneurysms, dot intraretinal hemorrhages, hard exudates, or cotton wool spots in the retina. As the disease progresses, there is an increasing distortion and leakage of retinal blood vessels. These detectable abnormalities result from vascular dysregulation and the detachment of endothelial cells from pericytes on histopathology. The leakage of retinal capillaries allows the passage of inflammatory mediators, proteins, and other blood contents into interstitial space, leading to the development of diabetic macular edema (DME) 23. In the severe NPDR stage, dysfunctional microvessels fail to supply adequate blood to retina, triggering oxidative stress reaction and compensatory angiogenesis. New blood vessels, accompanied by fibrotic tissue, grow along the inner surface of the retina and even extend into the vitreous. These fibrotic vessels may contract and cause tractional retinal detachment, eventually resulting in permanent vision loss 24.

**Figure 2:**
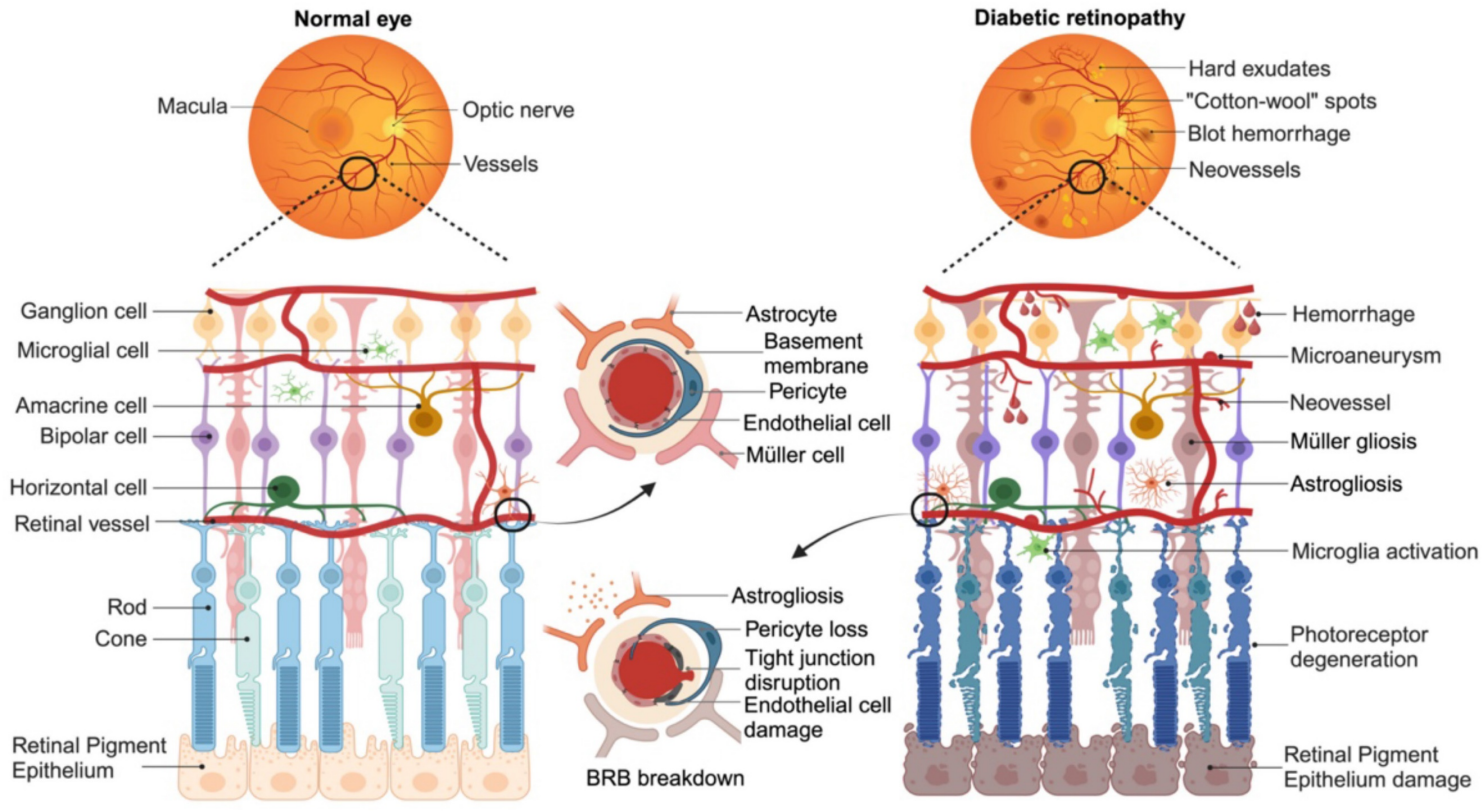
Schematic representation of key pathogenetic events in retina during the development of DR. The diagram illustrates the major changes that occur from a normal state (left) to an advanced stage of DR (right). Retina is composed of a complex network of capillary plexuses and multiple layers of cells. In DR, there are various vascular abnormalities, including thickening of basement membrane, endothelial injury leading to disruption of the tight junctions, and loss of pericyte. These changes result in the formation of microaneurysms, dot hemorrhages, hard exudates, and “cotton wool” spots. Compensatory neovascularization occurs as a response to the damaged vasculature. Additionally, dysfunction of glial cells (Müller cells or astrocytes) and activation of microglia contribute to the progression of the disease. Furthermore, degeneration of ganglion cells and photoreceptor cells (cones and rods) exacerbates the loss of vision associated with DR. (Figure created with BioRender.com).

Three layers of capillary plexuses embed in the retina offer needed nutrition and maintain the retinal homeostasis. Inner blood-retina barrier (BRB) composed by the tight junction of endothelial cells, podocytes, and glial endfeets. While the single layer of retinal pigment epithelium cells (RPEs) and Bruch's membrane (BM) constitute outer BRB that regulates the exchange of metabolites from the photoreceptor cells (cones and rods) with the underlying choroidal vessels. Both the inner and outer BRB act as barriers between the systemic circulation and the retina 25. More than a network of blood vessels, retina with multilayer structure is composed of neurons (photoreceptors, horizontal cells, bipolar cells, amacrine and ganglion cells) and glial cells (astrocytes, microglia, and Müller cells) 26, accounting for approximately 95% of the tissue 27. Essential for the BRB formation, glial cells dysregulate after microvascular damage, including Müller cells or astrocyte gliosis and microglia activation. Activated microglia, as the primary resident innate immune cells, can present a pro-inflammatory (M1) state and release various inflammatory cytokines, such as interleukin-1β (IL-1β), interleukin-6 (IL-6), interleukin-8 (IL-8), and tumor necrosis factor-α (TNF-α) 28, further exacerbating microangiopathy. Glial dysfunction also disrupts homeostatic and metabolic support to neuronal cells and photoreceptors, contributing to neurons death and axon degeneration 29. These irreversible neuronal abnormalities directly contribute to vision loss in DR patients. Emerging basic and clinical studies have shown that neuronal disorder may develop before the clinical observation of microvascular lesions, even though DR is primarily diagnosed based on vascular abnormalities 30. Moreover, the American Diabetes Association defines DR as a specific neurovascular complication of diabetes 22, highlighting the importance of both vascular and neuronal pathology in the development of DR (Figure 2).

## 3. EVs in DR pathology and diagnosis

### 3.1 The impact of EVs on pathological progression of DR

**Figure 3:**
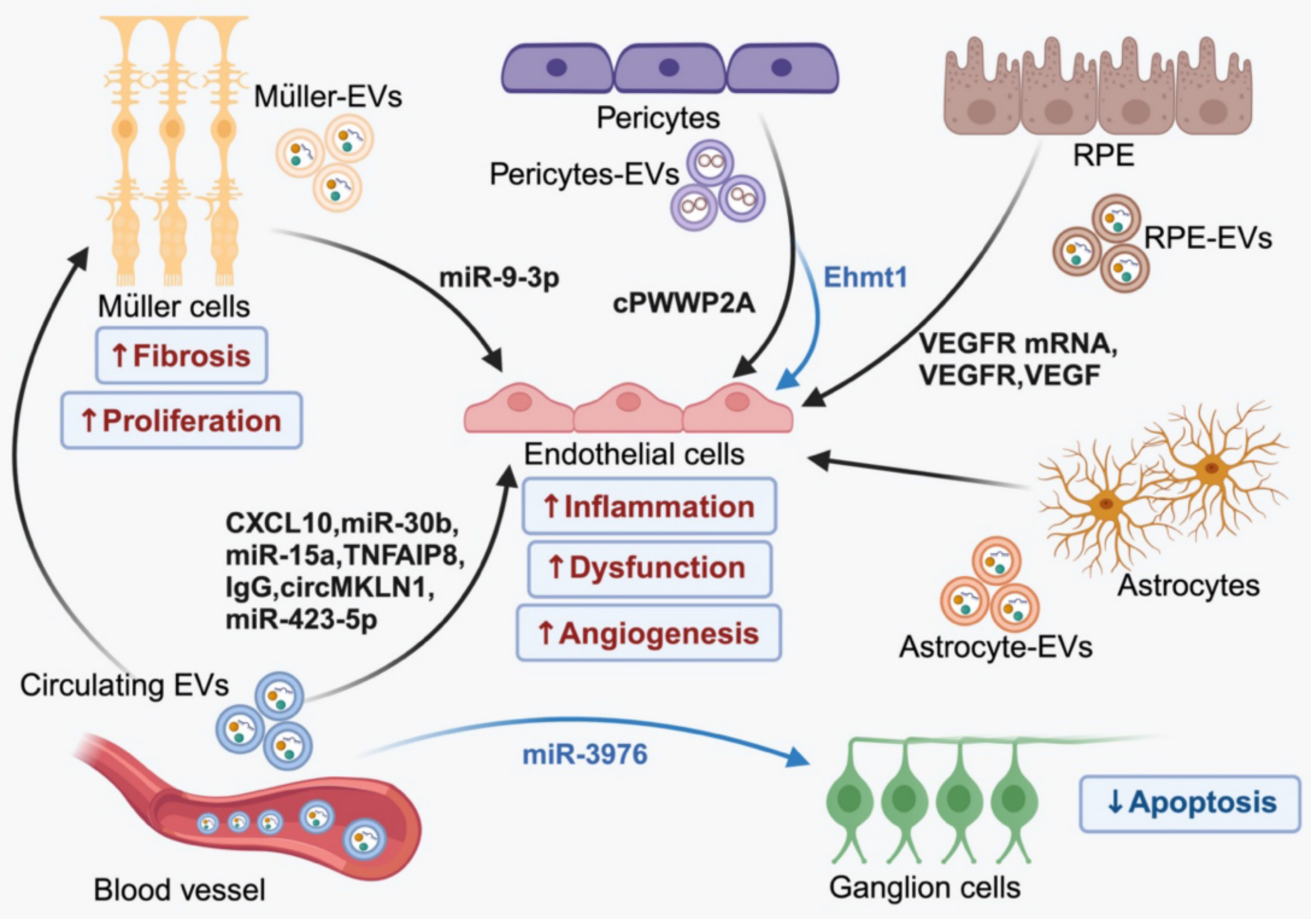
EV-mediated intercellular communication in the pathology of DR. In the context of DR, EVs derived from blood and a group of retinal cells contribute to the advancement of the diseases by facilitating the transfer of bioactive molecules between cells. Importantly, EVs originating from the same source can have both harmful and beneficial effects, which can be attributed to the different cargo they contain. (Figure created with BioRender.com).

Recent studies have highlighted the significance of EV-mediated endogenous intercellular communication in maintaining retinal cell function and homeostasis. In healthy retina, EVs released by photoreceptor cells are selectively internalized by Müller cells 31. Dysregulation of this EV communication process can exacerbate retinal degeneration 32. Additionally, disrupting EV biogenesis using GW4869, an exosome inhibitor, leads to altered EVs profiles (increased lEVs and reduced sEVs) in retinal ganglion cells, promoting neurodegeneration in a mouse model 33. In DR, persistent high blood glucose levels induce a cascade of pathological effects, including inflammation, oxidative stress, angiogenesis, and apoptosis 34. These pathological stimuli are likely responsible for the changes in cargo specificity and release of EVs. Recent studies in both experimental models and humans have observed elevated levels of EVs that correlated with inflammatory conditions 35 and neurodegenerative lesions 36. Wozniak et al. 37 disclosed that inflammasome activation contributes to caspase-1-mediated RILP cleavage, which can promote selective loading of miRNA cargos and secretion of EVs. In the retina, the activation of inflammasome triggers the release of EVs that encapsulate pro-inflammatory cytokines, such as interleukin-10. This is mediated by Gasdermin D, a terminal inflammasome component pyroptotic pore-forming protein, resulting in the propagation of inflammatory cascades 38. Additionally, study has shown that oxidative stress can lead to the secretion of enhanced numbers of basolateral EVs by RPEs containing changed protein contents related to epithelial barrier integrity, even though tight junction integrity remains unaffected 39. Although the exact mechanism of pathological EVs formation is still being investigated, substantial evidence suggests the involvement of disordered EVs in the development of DR, which contribute to ocular dysfunction by delivering special signals among retinal cells (Figure 3).

#### 3.1.1 The role of EVs in vascular lesions in DR

At the early stage of DR, vascular abnormities such as hemorrhage and neovascularization occur, characterized by thickening of the basement membrane, endothelial dysfunction, disruption of the tight junctions, pericyte loss, and the formation of acellular capillaries. Several studies have reported the involvement of aberrant circulating EVs in vascular damage during the development of DR. Jung et al. 40 suggested that elevated levels of endothelial cells (ECs)- derived EVs in peripheral blood (PB) are associated with vasculopathy of DR patients. Research indicates that circulating EVs originating from platelets, leukocytes, and endothelial cells in individuals with diabetes transport pro-angiogenic factors and proinflammatory cytokines, contributing to vascular complications such as retinopathy 41, 42. For example, in condition of hyperglycemia, EVs derived from platelet-rich plasma (PRP) can induce retinal endothelial injury by transferring CXCL10 and upregulating the TLR4 signaling pathway 43. Increased IgG-laden EVs in plasma can activate the classical complement pathway and promote membrane attack complex (MAC) deposition, accelerating vascular damage associated with DR 44, 45. Additionally, miR-15a enriched EVs in the plasma of DM patients are believed to be mainly produced in pancreatic β-cells, which can travel through the bloodstream, leading to retina damage by inducing oxidative stress 46. The serum EVs from individuals with DR have been found to exhibit elevated levels of circMKLN1. This circRNA specifically targets the miR-26a-5p/Rab11a axis, leading to the induction of retinal microvascular dysfunction 47. Moreover, these pathogenic EVs in PB also promote thrombosis in DR patients 48, thereby increasing the risk of thrombo-embolic DR.

Retinal vessel dysfunction in DR leads to increased leukostasis and thrombosis, resulting in local blockage of capillaries and hypoxia. This process triggers a series of adaptive responses, with one of the direct consequences being the activation of hypoxia-related factors. These factors, including hypoxia-inducible factor-1alpha (HIF-1α), VEGF, erythropoietin (EPO) and EPO receptor (EPOR), progressively increase in a time dependent manner in DR process 49, whose upregulation plays a crucial role in the permeability of microvessels and BRB break down 50. Meanwhile, these diabetes-related stress disrupts vascular stability via the EV mediated interaction between vascular endothelial cells and pericytes 51. Under high glucose (HG) condition, there is increased level of circRNA cPWWP2A in pericytes, which can be transferred from pericytes to ECs through EVs. The aberrant expressed cPWWP2A acts as a sponge for miR-579 and promotes the expression of angiopoietin 1, occludin, and SIRT1, leading to vascular cell damage and microvascular dysfunction 52. However, overexpressed vesicular-circEhmt1 from hypoxia-induced pericytes exhibits protective effect in ECs against HG-induced injury by suppressing NLRP3-mediated inflammasome formation 53. In the absence of therapeutic intervention, vascular damage becomes inevitable during the progression of DR, despite the therapeutic potential of pericyte-derived EVs in communication with ECs.

Neovascularization occurs as a compensatory response to severe vessel damage following retinal ischemia or infarction. These fragile new vessels, with abnormal architecture, are susceptible to bleeding and protrude into the vitreous body where they eventually anchored by fibrovascular tissue. Recent investigations into the mechanisms involved in abnormal retinal angiogenesis have identified several novel molecular players, including ADAM10 54 and the Netrin-1-CD146-Gαi1/3 axis 55. However, angiogenic factors, especially VEGF, are considered to be the primary drivers of disease progression in DR. Hyperglycemia and oxidative stress stimulate the secretion of EVs loaded with VEGF, VEGF receptors (VEGFR), and VEGFR mRNA by RPEs, promoting neovascularization in PDR 56-58. These pathological EVs not only deliver VEGF-related molecules directly but also induce the activation of VEGF signaling in ECs. In case of PDR, elevated levels of miR-9-3p are transferred to retinal endothelial cells through EVs derived from Müller glia. This transfer activates S1P1/AKT/VEGFR2 pathway, exacerbating abnormal angiogenesis 59. Additionally, the increased tumor necrosis factor-α-induced protein 8 (TNFAIP8) and miR-30b in plasma EVs have been revealed to promote proliferation in human retinal microvascular endothelial cells 60, 61, making them crucial mediators of angiogenesis. Recent research has also discovered low levels of lncRNA PPT2-EGFL8 and elevated miR-423-5p in plasma EVs derived from PDR patients. Reduced PPT2-EGFL8/miR-423-5p binding activates hypoxia-induced peroxisome proliferator-activated receptor-β/δ (PPARD)/angiopoietin-like 4 (ANGPTL4) signaling pathway, leading to diabetes-related neovascularization and retinal vessel breakage 62. Luo et al. 63 made an interesting discovery regarding a cluster of Tsp-1^+^ microglia cells that mitigate retinal neovascularization in mice with oxygen-induced retinopathy (OIR). Mechanically, these microglial cells release EVs containing Tsp-1, which are then transported to ECs and inhibit their migration and proliferation by regulating the miR-27a-5p/Smad3 axis. This finding offers a potential target for inhibiting pathologic neovascularization in DR.

#### 3.1.2 Role of EVs in neuronal and glial cell damage

Vessels, neurons, glia, and retina-resident immune cells constitute the retinal neurovascular unit. Retinal vasculature supplies essential nutrients to neural tissue. While neural and glial cells can regulate vascular cells as feedback, ensuring the normal function of the retina 64. Disrupted BRB in DR can jeopardize neurovascular homeostasis, leading to dysfunction of neural and glial cells and eventual vision loss. Loss of tight junction allows circulating EVs to cross the damaged endothelial barrier and enter interstitial space 65, which may influence retinal cell viability. In vitro, studies have demonstrated that EVs isolated from the PRP of diabetic rats upregulate both the proliferation and fibrogenic activity of retinal Müller glia cells though the Yes-associated protein (YAP)/PI3K/Akt pathway 66. Notably, although overexpression of miRNA-3976 promotes the apoptosis of retinal ganglion cells 5 (RGC-5), EVs carrying upregulated miRNA-3976 in plasma of DR patients have been found to increase proliferation and reduce apoptosis of RGC-5 cells, thus exerting a protective effect in vitro 67. Further in vivo studies are required to elucidate the impact of circulating EVs on retinal resident cells.

EVs from astrocytes (AD-EVs) has long been considered as the important participants in glia-neuron crosstalk, whose cargos are reported to be stimulus-dependent and exert pro-inflammatory or neuroprotective effects based on different context 68, 69. Under physiological conditions, AD-EVs play a role in transferring bioactive molecules that contribute to neuronal survival and neurite outgrowth 70. However, under pathological conditions, AD-EVs can exacerbate inflammatory in neural cells 71 and even trigger peripheral immunological response when released into circulation 72. These findings indicate a potential relationship between neurodegeneration and EVs derived from activated astrocytes in DR, given the oxidative stress and inflammation induced by chronic hyperglycemia and hypoxia 34, 73. Moreover, retinal astrocytes under oxidative stress exhibit increased levels of autophagy and release EVs that promote the proliferation and migration of ECs 74, showing the potential to promote neovascularization.

Overall, numerous studies have demonstrated the involvement of EVs in various pathologies associated with DR, including blood vessel damage, apoptosis of retina neurons and dysfunction of glial cells. EVs derived from retinal cells, vitreous and blood transport physiological or pathological signals through the change of amounts or special cargo contents, thus mediating biological functions of neighbor cells and even others located further away. The broad participation of EVs in DM and other connected complications have been reported besides DR 75. Given the systematic dysregulation in diabetes, it is important not to overlook the EV-mediated communication between the retina and other remote organs. As mentioned above, numerous studies have identified abnormal EVs circulating in the bloodstream that can damage retinal vascular endothelial cells. However, the organ-specific origins of these circulating EVs remain unclear. Furthermore, the existence of BRB increases the difficulty for the delivery of circulating EV into retina. Abundant evidence indicates that EVs have the ability to traverse biological barriers, including blood-brain-barrier (BBB) in the central nervous system, which is similar to BRB 76. Multiple potential pathways for EVs to cross the endothelial barrier are currently being extensively explored 77, shedding light on the possibility that EVs shuttling between the bloodstream and retina may precede BRB leakage. In addition to inner BRB composed of ECs, the outer BRB formed by RPEs further complicates EVs transport within the retina. A comprehensive understanding of ocular structures will expand research and application possibilities for EVs in retinal disorders.

### 3.2 EVs as diagnostic and prognostic biomarkers in DR

Growing evidence suggests that the regular fluctuation of EV quantities and cargos holds significant potential for providing predictors toward clinical diagnosis and prognosis in patients with DR. For example, a higher level of plasma EV numbers has been observed in PDR patients compared to those with NPDR or NDR 48. The number of platelet-derived EVs and monocyte-derived EVs in plasma elevate with the progression of the DR 78, 79. Moreover, an increased number of specific metabolites has been identified in the plasma EVs with progressive DR, suggesting the value of EVs contents in reflecting the progression of DR 80 (Table 1). The exploration of EVs as telltale indicators of DR is gaining more attention.

#### 3.2.1 Protein cargos of EVs for the diagnosis of DR

In plasma of patients with DR, an observed correlation has been found between the increased number of CCR5-positive (CCR5^+^) EVs and the progression of DR 81. Systematic analysis of proteomic profiles conducted by Xiao et al. 60 revealed the elevated expression of TNFAIP8 in the EVs derived from both plasma and vitreous humor in DR patients, suggesting that TNFAIP8 in plasma EVs could serve as a suitable biomarker of DR. Similarly, proteomic analysis of EVs derived from vitreous humor in the PDR patients has identified a series of differentially expressed proteins, including 4 upregulated proteins (immunoglobulin lambda-like polypeptide 1(IGLL1), triosephosphate isomerase 1 (TPI1), lactate dehydrogenase A (LDHA), apolipoprotein B(ApoB)) and 6 downregulated proteins (apolipoprotein M (ApoM), ficolin 3 (FCN3), aldolase C (ALDOC), serum amyloid protein (SAA) 2-4, insulin-like growth factor binding protein, acid-labile subunit (IGFALS), hyaluronan binding protein 2 (HABP2)), which show promise as candidates for PDR diagnosis. LDHA, FCN3, ApoB and ApoM have been further proved to be able to distinguish PDR patients from the normal controls in a cohort study with area under the curve (AUC) > 0.75 (0.777, 0.820, 0.910, 0.840) 82. Interestingly, EVs present in urine also hold potential for identifying DR. In a study conducted by Mighty et al. 83, specific expression of junction plakoglobin (JUP) was revealed in EVs from the urine of DR patients, but not in those from healthy donors or diabetic patient without DR. Due to its non-invasiveness and easy availability, urine is considered as attractive source for biomarkers compared to vitreous humor, plasma, or serum. Therefore, further exploration of the application of urine EVs for DR diagnosis is warranted.

#### 3.2.2 RNA cargos of EVs for the diagnosis of DR

The dysregulated miRNAs shuttled by EVs have been extensively recognized to offer diagnostic and/or prognostic information for type 2 diabetes mellitus (T2DM) and related cardiovascular complications 84, whose levels can also reflect the status of DR. Several studies focus on the alterations of circulating EV-miRNAs in biofluids from diabetic patients with/without retinopathy, as well as normal controls. Mazzeo et al. 85 discovered a specific miRNA signature in circulating EVs isolated from diabetic patients with retinopathy, which differed from both patients without complications and healthy controls, with 11 differentially expressed miRNAs identified. Upon further validation, they found that miR-150-5p, miR-21-3p, and miR-30b-5p not only involved in the pathological process of DR, but also play hold potential as biomarkers 86. Another study showed that elevated expression of EV-miR-15a in plasma is correlated with initial retinal damage, suggesting its potential application in the early prevention of DR in patients with T2DM 87. Similarly, miR-3976, miR-431-5p, and miR-26b-5p derived from serum EVs have been reported to be up-regulated in patients with DR 67, 88, 89, indicating their potential utility in monitoring the progression of DR. Tears, a valid source of biomarkers for ocular diseases, show aberrant proteomics or metabolomics alterations in DR 90, 91. Moreover, EVs from tear samples have been isolated and performed transcriptomic analysis, which disclosed the upregulated miR-145-5p, miR-214-3p, and miR-9-5p and the downregulated miR-146a-5p, miR-31-5p, and miR-96-5p in diabetic patients with retinopathy compared with the control 92. These dysregulated EV miRNAs offer solid indication for the disease-specific biomarker screen in DR diagnosis.

As the important regulator of miRNA, circRNA and lncRNA in DR patients also change specifically in DR pathogenesis. For instance, in DR patients there are increased levels of circMLKN1 in EVs from serum 47 and cPWWP2A in EVs secreted by pericytes 52, both of which mediate microvascular damage under diabetes-related stress. Xu et al. 62 separated serum EVs of healthy controls, DM individuals with NPDR, PDR, or without DR, and performed total RNA deep sequencing. They found aberrantly decreased levels of lncRNA PPT2-EGFL8 and significantly high expression of miR-423-5p due to competing endogenous RNA (ceRNA) mechanism. Another study analyzed lncRNAs in plasma EVs from individuals with T2DM and comorbid DR and identified lncRNA DLX6-AS1, PRINS, and FAM190A-3 as potential predictor of DR, with separate AUC values of 0.658, 0.798, and 0.603. Notably, the combination of DLX6-AS1 and PRINS obtained an AUC of 0.813, which even reached 0.860 in males 93.The construction of an EV-related ceRNA network in DR has revealed a series of aberrant expressed lncRNAs, miRNAs, and mRNAs 94, highlighting the broad prospects of vesicular RNAs in diagnosis applications.

The potential of EVs as non-invasive liquid biopsy has been further supported by clinical phase 1 trails conducted in various diseases, including in the diagnosis of DM 95. As for DR, there is also promising evidence suggesting that EVs found in tear, vitreous humor, serum, plasma, and urine hold great potential as diagnostic and prognostic markers. However, in addition to comparing EVs between healthy individuals and patients with varying degrees of DR, demographic factors such as age, race, and sex should also be considered in clinical cohort studies, as they can significantly influence EV characteristics 96. Furthermore, the availability of commercial kits and machines for EV capture and analysis plays a crucial role in facilitating the clinical translation of EV biomarkers. For instance, Pan et al. 80 reported that Fe_3_O_4_@TiO_2_ microbeads can efficiently and reversibly capture EVs from plasma (100 μL) within 20 min, compared to the hours required by traditional ultracentrifugation method. Another example is the iTEARS system developed by Hu et al. 92, which enables rapid isolation of EVs from small teardrop samples (less than 10 μL) with high yield and purity in only 5 min. These innovative EVs isolation methods have been successfully validated in DR patients, providing valuable insights into the pathological changes of EVs and highlighting the significant potential of EV biomarkers for clinical applications.

## 4. EV-based therapy in DR

**Figure 4:**
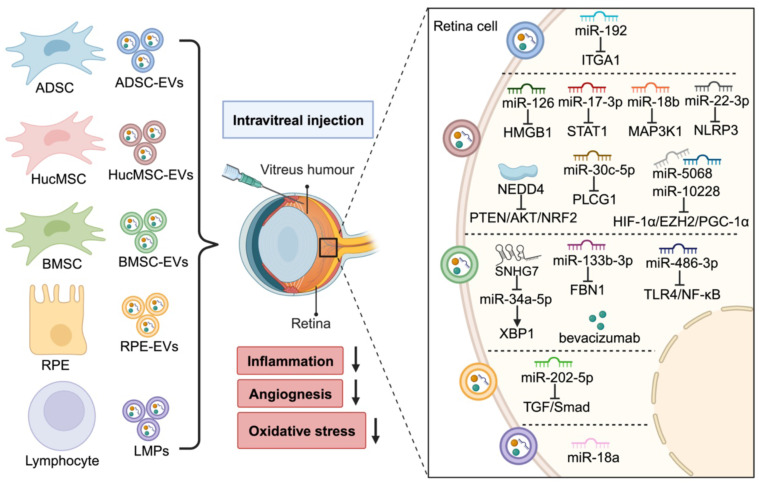
Therapeutic effects of EVs in DR. After intravitreal injection, MSC-EVs, RPE-EVs, and LMPs transfer various cargos and could alleviate DR progress by reducing inflammation, oxidative stress, and angiogenesis. Some research also loads the drug (e.g., bevacizumab) into EVs to improve the therapeutic effect. (Figure created with BioRender.com).

Laser photocoagulation, intraocular administration of steroids and anti-VEGF agents, and vitrectomy are the most common used clinical treatment for DR, aimed at minimizing vision loss 97. However, the limited efficacy and unavoidable side effects of these therapies remain unresolved 98. Therefore, there is an active demand for new therapeutics. Emerging evidence suggests that EVs derived from various cell types, including MSCs, retinal cells, and immune cells, hold great promise in DR therapy (Figure 4).

### 4.1 MSCs-derived EVs

Due to their self-renewal and differentiation properties, MSCs are considered as promising candidates for tissue repair and regenerative medicine. EVs secreted from MSCs (MSC-EVs) have been widely studied as therapeutic molecules in both in vitro cell models and preclinical animal models of diabetes and its complications, including DR 99 (Table 2). Our previous research has shown that EVs from human umbilical cord MSCs (hucMSC-EVs) can reduce blood glucose levels, partially reverse peripheral insulin resistance, and alleviate β-cell destruction in a streptozotocin (STZ) induced T2DM rat model 100. More recently, we have also found that intravitreal injection of hucMSC-EVs effectively improves retinal function, reduce retinal oxidative stress, apoptosis, inflammation, and angiogenesis in both leptin gene deficient db/db mice and STZ-induced diabetic rats. Mechanistically, miR-5068 and miR-10228, and neuronal precursor cell-expressed developmentally downregulated 4 (NEDD4) carried by hucMSC-EVs can modulate HIF-1α/EZH2/PGC-1α and PTEN/AKT/NRF2 signaling pathway separately 101, 102. In the study by Zhang et al. 103, hucMSC-EVs treatment was shown to inhibit hyperglycemia induced retinal inflammation in both in vivo and in vitro DR models by transporting miR-126, a suppressor of the HMGB1 signaling pathway. Similarly, several studies have reported that hucMSC-EVs carrying specific miRNAs, such as miR-17-3p 104, miR-18b 105, miR-30c-5p 106, and miR-22-3p 107, have the ability to ameliorate inflammatory response and oxidative injury in DR mouse models via targeting STAT1, MAP3K1, PLCG1, and NLRP3 respectively. Moreover, intravitreal administration of hucMSC-EVs has also been shown to protect retinal ganglion cells from apoptosis, thereby reducing neurodegeneration (DRN) and improving the retinal structure in a DR rat model 108.

Bone marrow mesenchymal stem cells (BMSCs) are considered as a safe and effective treatment option for DR 109, and their EVs also show great potential for therapy. Li et al. 110 found that EVs from BMSCs (BMSC-EVs) can reduce oxidative stress and inflammation and protect the viability of HG-treated Müller cells via delivering miR-486-3p that repress TLR4/NF-κB signaling pathway. In HG-stimulated mouse retinal microvascular endothelial cells (mRMECs), BMSC-EVs loaded with miR-133b-3p have been demonstrated to reduce pathological angiogenesis and oxidative stress by targeting FBN1 111.

Similar results have been observed in a STZ induced DR rat model, whose retinal injury was alleviated by BMSC-EVs through the suppression of the Wnt/β-catenin signaling pathway, resulting in reduced oxidative stress, inflammatory response, and angiogenesis 112. The BMSC-derived vesicular lncRNA SNHG7 could suppresses HG-triggered endothelial-to-mesenchymal transition (EndoMT) and tube formation in human retinal microvascular endothelial cells (hRMECs) by targeting miR-34a-5p/XBP1 axis 113. Furthermore, EVs extracted from adipose mesenchymal stem cells (ADSC-EVs) exhibits similar benefits as BMSC-EVs in DR therapy. A study by Gu et al. 114 revealed that intravitreal administration of ADSC-EVs delivered miR‐192, which negatively regulates ITGA1, thus delaying the events of inflammation and angiogenesis. In another study of Reddy et al. 115, BMSC-EVs loaded with bevacizumab, an anti-VEGF antibody commonly used to treat DR, reduced retinal leakages, leukocytosis, and cell apoptosis in a rat model of DR. Moreover, the combination of EVs and bevacizumab prolongs the therapeutic effect from one month to two months, indicating the diverse application possibilities of MSC-EVs for DR treatment.

### 4.2 Retinal cells and immune cells derived EVs

RPEs are crucial for maintaining the integrity of the outer BRB and normal vision 25. Despite that RPEs undergo pathological changes during the progression of DR, it has been suggested that RPEs derived EVs (RPE-EVs) may have a therapeutic role in DR. For example, EVs isolated from HG-stimulated RPEs are rich in miR-202-5p and can inhibit EndoMT of ECs under HG condition by targeting TGF/Smad pathway 116. In addition, another research indicates that bevacizumab, when administered intravitreally, can be taken up by RPEs and then released though EVs, offering a novel mechanism by which therapeutic antibodies exert beneficial effects on distant non-target cells 117. Photoreceptor cells are located in the outer retina and adjacent to RPEs, the later also have a significant impact on maintaining the normal structure and function of photoreceptor cells. Both RPEs and photoreceptor cells contribute to the progression of DR 118. A recent study suggests that overexpression of Thioredoxin alleviates diabetics-induced degeneration of photoreceptor cells by mediating autophagy and secreting EVs, which can then be phagocytosed by RPEs and enhance their biological function during DR in vitro 119. The EV-mediated communication between RPEs and photoreceptor cells may establish a beneficial feed-forward cycle in DR therapy.

Human T lymphocytes derived EVs (LMPs) have been reported to possess strong anti-angiogenic abilities in several ocular diseases 120-122. LMPs-mediated inhibition of choroidal neovascularization has been proved to depend on the integrity of RPEs, which express increased levels of antiangiogenic factors (pigment epithelium-derived factor) and trigger the apoptosis of ECs by activating neurotrophin receptor p75 120. In the mouse model of laser-induced choroidal neovascularization, LMPs have been shown to target macrophages and prevent their proangiogenic polarization, leading to a downregulation of proangiogenic factors (e.g., VEGFa, interleukin-10) and an upregulation of antiangiogenic factors (e.g., interleukin-12, thrombospondin-1) 121. The reduced pathological retinal neovascularization has also been observed in a mouse model of OIR after intravitreal injection of LMPs. Specifically, LMPs inhibit the proliferation of Müller cells and decrease VEGF expression, thereby attenuating the infiltration of proangiogenic macrophages 122. A study by Yang et al. 123 analyzed the expression profiling of RNAs in LMPs and found selective enrichment of miR-18a, which inhibited retinal neovascularization in both in vitro and in vivo models of OIR. This discovery aids in identifying optimal targets and effective treatments to prevent abnormal neovascularization in DR.

Improving the circulation half-lives of anti-DR drugs is crucial to minimize side effects caused by frequent intraocular administration 124. Utilizing EVs as a drug delivery platform is advantageous due to their specifical membrane-bound structure. The combination of drugs and EVs exerts a prolonged and better treatment benefit than either drug or native EVs administration alone 115. However, when compared to conventional drugs, very few studies have reported the cellular uptake and vitreous pharmacokinetics of EVs after injection into retina. In a rat model of retinal ischemia, functional recovery and reduced neuro-damage can be observed after injection of BMSC-EVs into the vitreous humor 24h. These EVs have been found to be taken up by retinal neurons, ganglion cells, and microglia, and remain in the vitreous humor for four weeks 125. However, another study found that the lifetime of EVs in rat vitreous humor is only 2.5 days. Moreover, EVs are mainly internalized by cells in the outer nuclear layer, with varying retention times in different cell types 126. Thus, enhancing vitreous retention and targeting ability may be the key point for improving the therapeutic efficiency of EVs in DR. In a current paper by Bao et.al 127, BMSC-EVs were encapsulated within polymeric microcapsules, allowing for controlled release as the capsules gradually degrade. These microencapsulated EVs not only show enhanced biological stability but also demonstrate sustained release for more than one month following intravitreal injection in a mouse model of retinal ischemia-reperfusion injury. In EV therapy, in addition to being loaded with therapeutic cargos and combined with biomaterials, EVs membranes can also be modified to improve targeting properties and tracing abilities 128. These engineering strategies address the limitations of native EVs, thereby expanding their potential as the next generation of nanomedicine for DR.

## 5. Perspectives and challenges

Although significant progress has been made in understanding the biological features of EVs and their specific roles in diabetic retina damage, many challenges impede the development of EV research, one of which is the heterogeneity of vesicles. Most of the EVs in published studies consist of mixed subpopulations and may even be accompanied by non-vesicular contaminants due to the technical barriers associated with standard EV isolation protocol 129, 130. Conventional methods for isolating EVs, such as ultracentrifugation, ultrafiltration, and precipitation, often result in co-separation of lipoproteins or other contaminants that have similar physicochemical properties to EVs. These methods may even disrupt the structural and biological integrity of EVs due to external force 131. While immunoaffinity capture technology offers the potential to isolate a particular subpopulation of EVs based on specific protein markers, its limited small-scale production cannot be ignored 132. The “cocktail strategy”, which involves combination of multiple EV extraction protocols, is being widely researched to achieve high-purity and high-yield EV isolation in a shorter time frame 95. Notably, advancements in technology have enabled the detection and analysis of EVs at the single-vesicle level, offering valuable insights into the study of specific EV subtypes 133. Furthermore, thorough understanding of the complex origins, destination, and temporal dynamics of EVs in DR is limited by deficient in endogenous EV tracking system and imaging techniques that can operate across different scales. Recently, researchers have successfully constructed and utilized three-dimensional models of the retinal vasculature to investigate hyperoxia-induced vascular obliteration in a mouse model of OIR 134. This achievement highlights the potential for visualizing EVs and elucidating their contributions within the three-dimensional structure of the retina, thereby advancing our understanding of DR mechanisms, and identifying potential biomarkers.

**Figure 5:**
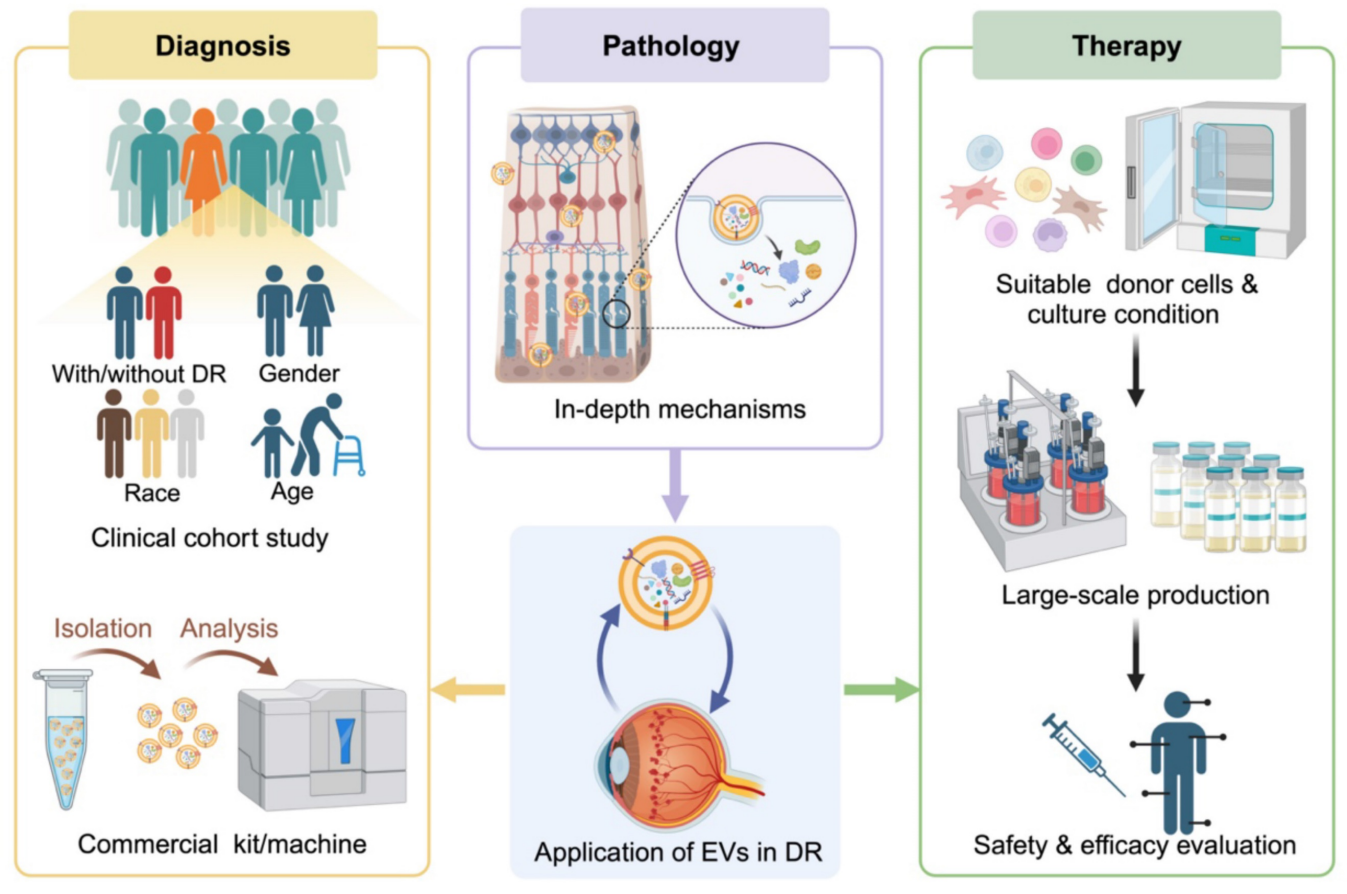
The potential future development of EVs as biomarkers and therapy agents in DR. Continued advancements in basic research will enhance our understanding of the involvement of EVs in the progression of DR, opening avenues for the development of new targets for disease diagnosis and treatment. However, there are still several key issues that require further exploration before the biomedical application and clinical translation of EVs can be realized.

Given the inherent therapeutic advantages of native EVs, which can be further enhanced through engineered modifications 135, there is considerable anticipation for the clinical application of EVs as therapeutic agents for DR. However, there are still some potential issues that need to be addressed. Firstly, EVs derived from donor cells under different condition may possess distinct biological features, making it difficult to maintain the stability of treatment effects. As mentioned earlier, BMSC-EVs are generally considered to alleviate retinal damage. While EVs from BMSC cultured in diabetic-like condition (HG and/or hypoxia) have been shown to promote BRB permeability and angiogenesis in vitro 136, 137. Therefore, more efforts should be made to explore the most suitable origin and culture condition for parental cells of therapeutic EVs. To meet the requirements for clinical administration, technologies for subsequent large-scale production of EVs, such as bioreactors that contain a dynamic monitoring system and maximized cell culture surface, are also popular directions worth investigating 138. Secondly, the complex nature of bioactive molecules in EVs poses challenges in fully understanding their functions. The presence of unknown contents increases the risk of potential unintended side effects during clinical administration. A comprehensive safety evaluation is imperative prior to clinical administration. (Figure 5).

Taken together, this review summarizes the key characteristics of EVs and DR, and highlight the research advances of the emerging roles played by EVs in the context of DR. Accumulating evidence strongly supports the notion that EVs, acting as important mediators of intercellular and inter-tissue communication, are crucial participants in the onset and progression of DR. Moreover, their potential as biomarkers and therapeutic interventions for DR is increasingly recognized. With advancements in basic research, EVs will find broad applications in the diagnosis and treatment of DR and other diabetic complications in the future.

## Figures and Tables

**Table 1:** EVs as biomarkers for DR

Source of EVs	EV cargo sequencing (type)	EV cargos/markers	Levels	Comparison	Ref.
serum	yes (circRNA)	circMKLN1	↑	DR VS DM and HC	47
plasma, vitreous humor	yes (protein)	TNFAIP8	↑	DR VS DM and HC	60
serum	yes (total RNA)	lncRNA PPT2-EGFL8/ miR-423-5p	↓/↑	PDR VS NPDR, DM, and HC	62
serum	yes (miRNA)	miR-3976	↑	DR VS DM	67
plasma	no	GPIX^+^ EV counts	↑	DR VS DM	78
plasma	no	Annexin V^+^ and CD14^+^ EV counts	↑	DR VS DM	79
plasma	yes (metabolite)	N, N′-dicyclohexylcarbodiimide, 4-acetamidobutyric acid, Dl-2-aminooctanoic acid/ triethanolamine	↑	NPDR VS DM/ PDR VS NPDR	80
plasma	no	CCR5^+^ EVs counts	↑	moderate and severe NPDR VS mild NPDR	81
vitreous humor	yes (protein)	IGLL1, TPI1, LDHA, ApoB/ ApoM, FCN3, ALDOC, SAA2-4, IGFALS, HABP2	↑/↓	PDR VS HC	82
urine	yes (protein)	JUP	↑	DR VS DM and HC	83
plasma, serum	yes (miRNA)	miR-150-5p/miR-21-3p, miR-30b-5p	↓/↑	DR VS DM and HC	85, 86
plasma	no	miR-15a	↑	DM with retinal damage VS HC	87
serum	yes (miRNA)	miR-431-5p,	↑	PDR VS DM and HC	88
serum	no	miR-26b-5p	↑	DR VS DM and HC	89
tear	yes (miRNA)	miR-145-5p, miR-214-3p, miR-9-5p/ miR-146a-5p, miR-31-5p, miR-96-5p	↑/↓	DR VS HC	92
plasma	yes (lncRNA)	lncRNA DLX6-AS1/PRINS, FAM190A-3	↑/↓	DR VS DM	93

HC: healthy control; DM: DM without DR; VS: versus

**Table 2:** Therapeutic function of MSC-EVs in DR

Source of EVs	Therapeutic molecules	Animal models	EV dosage/per eye	Method of administration	Effect of EVs in vivo	Clinical / pre-clinical object	Cell models	Effect of EVs in vitro	Ref.
hucMSCs	NEDD4	STZ-induced diabetic rats	1×10^6^ particles	IVT	attenuate retinal apoptosis and oxidative stress	pre-clinical object	HG-treated RPEs	promote cell proliferation, reduce oxidative damage	101
hucMSCs	miR-5068, miR-10228	STZ-induced diabetic rats and db/db mice	1×10^7^ particles	IVT	alleviate retinal apoptosis, inflammation, and angiogenesis	pre-clinical object	HG-treated hRMECs	mitigate cell proliferation, migration, and tube formation	102
hucMSCs	miR-126	STZ-induced diabetic rats	50 μg (RNA concentration)	IVT	alleviate retina inflammatory response	pre-clinical object	HG-treated hRMECs	reduce NLRP3 inflammasome	103
hucMSCs	miR-17-3p	STZ-induced diabetic mice	50 μg	IVT	ameliorate retina inflammatory reaction and antioxidant injury	pre-clinical object	-	-	104
hucMSCs	miR-18b	STZ-induced diabetic rats	10, 20, and 40 μg/mL	IVT	reduce inflammatory response and vascular leakage	pre-clinical object	HG-treated hRMECs	attenuate cell inflammation and apoptosis	105
hucMSCs	miR-30c-5p	STZ-induced diabetic rats	50 μg	IVT	suppress retinal inflammatory response	pre-clinical object	HG-treated hRMECs	inhibit cell inflammation	106
hucMSCs	miR-22-3p	STZ-induced diabetic rats	1.5 × 10^9^ particles	IVT	alleviate retinal inflammation, improve histological morphology and BRB function	pre-clinical object	advanced glycation end-products-induced microglial cells	alleviate cell activation	107
hucMSCs	-	STZ-induced diabetic rats	4 μL	IVT	improve retinal structure, reduce neurodegeneration	pre-clinical object	-	-	108
BMSC	miR-486-3p	STZ-induced diabetic mice	-	-	-	-	HG-treated Müller cells	inhibit cell oxidative stress, inflammation, and apoptosis, promote proliferation	110
BMSC	miR-133b-3p	KK/Upj-Ay mice	-	-	-	pre-clinical object	HG-treated mRMECs	reduce cell oxidative stress, angiogenesis, proliferation, migration, and promote apoptosis	111
BMSC	-	STZ-induced diabetic rats	50 μg (Protein concentration)	IVT	inhibit retina oxidative stress, inflammation, and angiogenesis	pre-clinical object	-	-	112
BMSC	lncRNA SNHG7	-	-	-	-	-	HG-treated hRMECs	suppress cell EndoMT and tube formation	113
ADSC	miR‐192	STZ-induced diabetic rats	5, 10, 20, and 40 μg/ml	IVT	alleviate inflammatory response and angiogenesis	pre-clinical object	HG-treated hRMECs, Müller cells, and RPEs	inhibit RPEs apoptosis, Müller cells activation, and hRMEC proliferation	114

IVT: intravitreal injection; hucMSC: human umbilical cord MSCs; STZ: streptozotocin; BMSCs: bone marrow MSCs; mRMECs: mouse retinal microvascular endothelial cells; EndoMT: endothelial-to-mesenchymal transition; hRMECs: human retinal microvascular endothelial cells; ADSC: adipose MSC.

## Abbreviations

DR: diabetic retinopathy
DM: diabetes mellitus
EVs: extracellular vesicles
VEGF: vascular endothelial growth factor
MVs: microvesicles
MVB: multivesicular bodies
NVEPs: non-vesicular extracellular nanoparticles
sEVs: small EVs
lEVs: large EVs
MSCs: mesenchymal stem cells
NPDR: non-proliferative DR
PDR: proliferative DR
DME: diabetic macular edema
BRB: blood-retina barrier
RPEs: retinal pigment epithelium cells
BM: Bruch's membrane
ECs: endothelial cells
PB: peripheral blood
PRP: platelet-rich plasma
MAC: membrane attack complex
HIF-1α: hypoxia-inducible factor-1alpha
EPO: erythropoietin
EPOR: EPO receptor
HG: high glucose
VEGFR: VEGF receptors
OIR: oxygen-induced retinopathy
RGC-5: retinal ganglion cells 5
AD-EVs: EVs from astrocytes
BBB: blood-brain-barrier
CCR5^+^: CCR5-positive
AUC: area under the curve
JUP: junction plakoglobin
T2DM: type 2 diabetes mellitus
ceRNA: competing endogenous RNA
MSC-EVs: EVs secreted from MSCs
hucMSC: human umbilical cord MSCs
STZ: streptozotocin
BMSCs: bone marrow MSCs
mRMECs: mouse retinal microvascular endothelial cells
EndoMT: endothelial-to-mesenchymal transition
hRMECs: human retinal microvascular endothelial cells
ADSC: adipose MSC
LMPs: human T lymphocytes derived EVs
